# Megakaryocytic Differentiation Regulates the Permissiveness and Antiviral Response of the Megakaryocytic Erythroid Progenitor to Dengue Virus

**DOI:** 10.3390/ijms262211081

**Published:** 2025-11-16

**Authors:** Diego Sait Cruz-Hernández, Francisco Javier Sánchez-Peña, Marymar Cruz-Cruz, Darío de Jesús Guillén-Morales, Martha Cristina Castillo-Soriano, Elizabeth Cruz-Altamirano, Juan Alpuche, José Bustos-Arriaga, María de Los Ángeles Romero-Tlalolini, Honorio Torres-Aguilar, Juan Carlos Rodríguez-Alba, Sergio Roberto Aguilar-Ruíz

**Affiliations:** 1 Departamento de Biomedicina Experimental, Facultad de Medicina y Cirugía, Universidad Autónoma “Benito Juárez” de Oaxaca, Oaxaca 68120, Mexico; cruzdiegos@hotmail.com (D.S.C.-H.); darioguillenmor@outlook.com (D.d.J.G.-M.); criss9511279069@gmail.com (M.C.C.-S.); 2 Secretaria de Ciencia, Humanidades, Tecnología e Innovación, Facultad de Medicina y Cirugía, Universidad Autónoma Benito Juárez de Oaxaca, Oaxaca 68120, Mexico; kowalski-23@hotmail.com (F.J.S.-P.); cucm93@gmail.com (M.C.-C.); romerotlalolini@gmail.com (M.d.L.Á.R.-T.); 3 Laboratorio de Bioquímica, Facultad de Medicina y Cirugía, Universidad Autónoma Benito Juárez de Oaxaca, Oaxaca 68120, Mexico; cuae881108.fmc@uabjo.mx (E.C.-A.); jalpuche.fmc@uabjo.mx (J.A.); 4 Laboratorio de Biología Molecular e Inmunología de Arbovirus, Facultad de Estudios Superiores Iztacala, Universidad Nacional Autónoma de México, Estado de México 54090, Mexico; jose.bustos@iztacala.unam.mx; 5 Facultad de Ciencias Químicas, Universidad Autónoma “Benito Juárez” de Oaxaca, Oaxaca 68120, Mexico; qbhonorio@hotmail.com; 6 Unidad de Neuroinmunología y Neurooncología, Instituto Nacional de Neurología y Neurocirugía, Ciudad de México 14269, Mexico; juan.rodriguez@innn.edu.mx

**Keywords:** megakaryocytic differentiation, dengue virus, interferon beta, K562 cells, erythroid-megakaryocytic progenitor

## Abstract

Dengue virus (DENV) affects not only peripheral immune cells but also hematopoietic progenitors in the bone marrow, particularly megakaryocytic precursors, which contribute to the thrombocytopenia characteristic of the disease. In this study, we evaluated the relationship between the differentiation status of the megakaryocytic lineage and its permissiveness and antiviral response to DENV. Our results demonstrate that the erythroid–megakaryocytic precursor (K562 cells) was more permissive to DENV infection than megakaryoblasts, as evidenced by immunofluorescence, flow cytometry, and quantification of viral particles. The antiviral response in K562 cells peaked at three days post-infection, with maximal expression of genes associated with the type I interferon (IFN-I) pathway. In vitro-induced differentiation of K562 cells reduced the initial susceptibility to DENV and enhanced the expression of Toll-like receptor 3 (TLR3) and the type I interferon receptor (IFNAR1), accelerating and intensifying IFN-β secretion, and increasing the expression of OAS2 and IRF3. Furthermore, pretreatment of K562 cells with recombinant IFN-β significantly reduced viral replication from the first day post-infection. Collectively, these findings demonstrate for the first time that the differentiation status of erythroid–megakaryocytic progenitor critically shapes their antiviral response and underscore the central role of IFN-β in the early restriction of DENV infection.

## 1. Introduction

The dengue virus (DENV), named according to the new taxonomy ratified by the International Committee on Taxonomy of Viruses (ICTV) in 2023 as *Orthoflavivirus denguei* [1], belongs to the *Flaviviridae* family and the *Orthoflavivirus* genus. It is a positive-sense single-stranded RNA virus (+ssRNA) that encodes three structural proteins: envelope (E), capsid (C), and membrane precursor (prM) [2]. DENV is classified into four antigenically distinct serotypes (DENV1 to DENV4). It is estimated that more than 60 million cases and over 20,000 deaths occur annually across approximately 130 countries; however, these figures may rise due to increased human mobility and the impact of climate change [3], which heightens the risk of transmission to previously unexposed populations and facilitates the geographic spread of the virus [4].

Infection with any of the four DENV serotypes can cause dengue disease. Since 2009, the World Health Organization (WHO) has established revised classification criteria, dividing dengue into two main categories: dengue with or without warning signs and a severe form known as severe dengue [2,5].

In addition to fever and other systemic symptoms, dengue frequently induces hematological alterations, most notably thrombocytopenia—defined as a reduction in peripheral platelet count—which is considered a hallmark of infection [6]. This thrombocytopenia has been attributed not only to peripheral platelet destruction via immunopathological mechanisms [7] but also to a decrease in megakaryocyte numbers, resulting in insufficient platelet production in the bone marrow [8,9]. The detection of DENV in bone marrow aspirates [10], combined with evidence that bone marrow cells are highly permissive to DENV infection, strongly supports this hypothesis [10,11,12]. In this context, DENV infection exerts a significant impact on the bone marrow microenvironment and hematopoietic processes. Emerging evidence suggests that hematopoietic progenitor cells play a key role in dengue pathogenesis [13].

Among the various hematopoietic lineages affected in the bone marrow, the megakaryocytic lineage appears to be the most compromised [6,10,11,14]. Under physiological conditions, megakaryocyte maturation is a unique process characterized by endomitosis—repeated cycles of DNA replication without cytokinesis—resulting in polyploid cells. This differentiation is primarily regulated by thrombopoietin (TPO) [15]. The erythroid–megakaryocytic progenitor (EMP) represents an early bipotential stage of hematopoiesis capable of differentiating into either megakaryocytes or erythrocytes and is particularly permissive to DENV infection [16,17,18].

It has been proposed that the maturation state of megakaryocytic progenitors modulates their susceptibility to DENV infection. In cellular models, undifferentiated megakaryoblastic cells such as MEG-01 and K562 have demonstrated high permissiveness to DENV [19,20,21]. Furthermore, DENV has been shown to propagate efficiently in megakaryocytic precursor cells once infection is established and differentiation is induced with phorbol 12-myristate 13-acetate (PMA) [19]. However, when these same cells are first differentiated with PMA prior to infection, they become refractory to viral entry and replication [9]. Collectively, these findings suggest that megakaryocytic differentiation not only governs platelet maturation but also modulates cellular permissiveness to DENV.

Beyond susceptibility and permissiveness determined by differentiation status, the intrinsic antiviral response of megakaryocytic progenitors to DENV has also been explored. Studies have shown that these precursors express pattern recognition receptors (PRRs), including RIG-I (Retinoic Acid-Inducible Gene I), MDA5 (Melanoma Differentiation-Associated protein 5), and TLR3 (Toll-Like Receptor 3) [22,23,24], which are capable of inducing type I interferon (IFN-I) production and the expression of interferon-stimulated genes (ISGs) upon infection [21,25]. In this context, IFITM3 (Interferon-Induced Transmembrane Protein 3) has been identified as a key antiviral component in megakaryocytes and platelets, as its expression restricts DENV replication, while low levels of this protein correlate with increased clinical severity [25]. In cellular models such as MEG-01 infected with DENV-2, progressive activation of the *RIG-I/MDA5* pathway has been observed, accompanied by increased expression of *IFN-β* and *ISG15* [21], confirming that these progenitors are capable of mounting a functional antiviral response. Conversely, in K562 cells, DENV-2 infection significantly reduced reactive oxygen species (ROS) accumulation—an effect mediated by Nrf2 activation—which favors viral replication [19].

Taken together, the evidence suggests that DENV infection of megakaryocytic progenitors is influenced not only by their differentiation status but also by their intrinsic ability to activate interferon-mediated antiviral pathways. However, important questions remain regarding the response of more immature megakaryocytic precursors to DENV. Therefore, this study aims to evaluate the permissiveness and antiviral immune response of the erythroid–megakaryocytic progenitor to DENV, analyzing how cellular differentiation and activation of the interferon pathway modulate its capacity to either restrict or facilitate viral replication.

## 2. Results

### 2.1. The Erythroid-Megakaryocytic Precursor Is More Susceptible to DENV1 Infection than Megakaryoblasts

**Figure 1:**
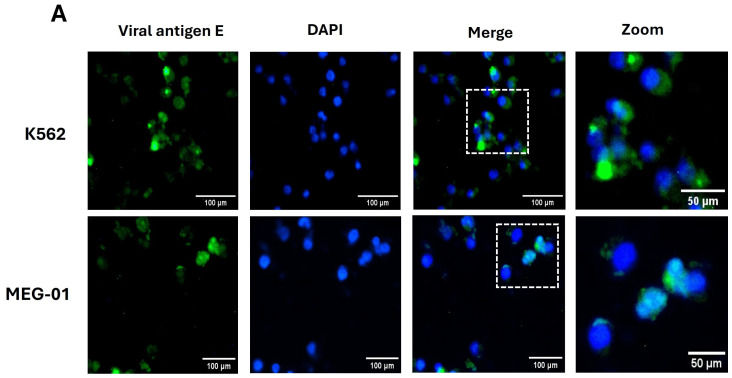

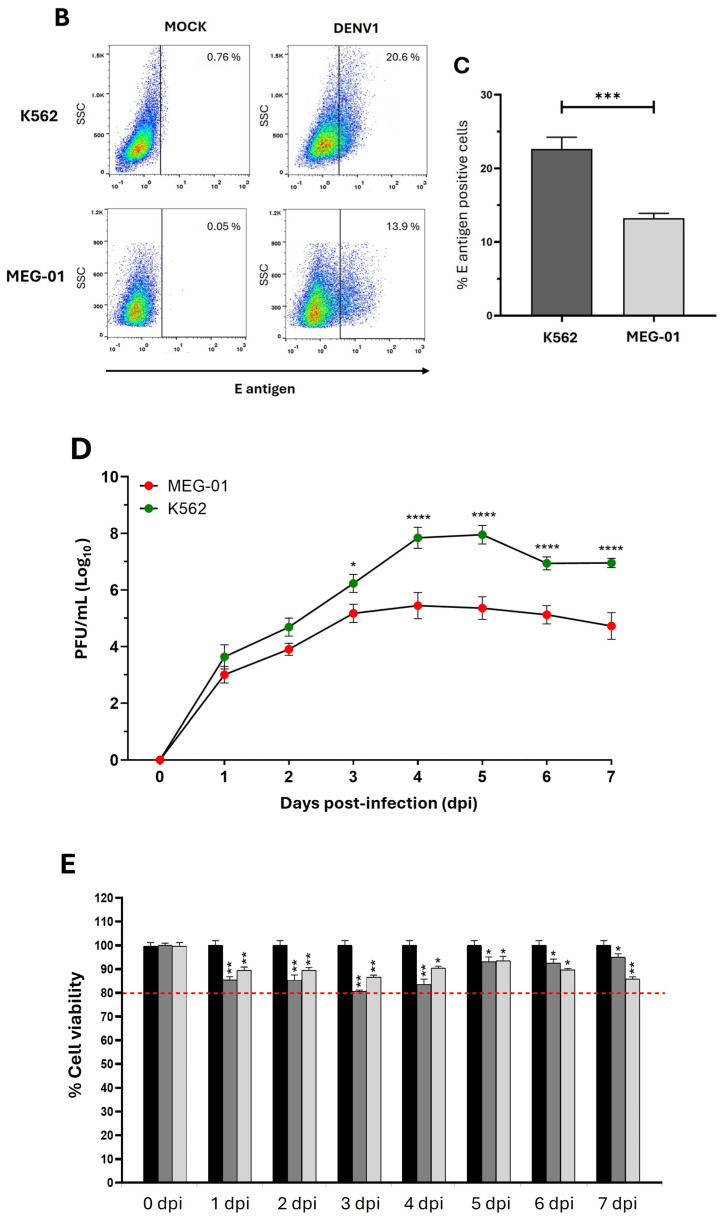
Megakaryocytic progenitors are differentially susceptible and permissive to DENV. (**A**) Representative immunofluorescence images of K562 and MEG-01 cells infected with DENV1 at a multiplicity of infection (MOI) of 0.5, showing detection of the envelope (**E**) antigen (green) and cell nuclei stained with DAPI (blue). Enlarged views highlight the intracellular localization of the antigen. (**B**) Representative flow cytometry dot plots showing E antigen detection in K562 and MEG-01 cells infected with DENV1 (MOI 0.5), compared to uninfected controls (Mock). (**C**) Percentage of E antigen-positive cells determined by flow cytometry at three days post-infection (dpi); statistical comparison between cell lines was performed using Student’s *t*-test. (**D**) Viral replication kinetics in cell supernatants up to seven dpi, expressed as plaque-forming units per milliliter (PFU/mL). (**E**) Cell viability of K562 (dark gray) and MEG-01 (light gray) up to seven dpi, assessed by MTT assay and expressed as a percentage relative to uninfected controls (black bars). In panels (**A**–**C**), a monoclonal antibody specific for the DENV E antigen was used. Significant differences in viral replication kinetics (**D**) and cell viability (**E**), were analyzed using a two-way ANOVA followed by Tukey’s test. Data are expressed as mean ± standard deviation from three independent experiments. Statistical differences are indicated as *p* < 0.05 (*), *p* < 0.01 (**), *p* < 0.001 (***), and *p* < 0.0001 (****).

To confirm the susceptibility of megakaryocytic progenitors to dengue virus serotype 1 (DENV1), infection was established in K562 and MEG-01 cell lines. The presence of the viral envelope (E) antigen was detected three days post-infection (dpi) by immunofluorescence (Figure 1A) and flow cytometry (Figure 1B,C), using monoclonal antibodies (mAbs). As previously reported by Clark et al. [18], both cell lines were susceptible to infection. Permissiveness was subsequently evaluated by quantifying infectious viral particles (Figure 1D) and assessing cell viability up to seven dpi (Figure 1E). These results confirm that both progenitor lines are susceptible to DENV1 infection; however, K562 cells exhibited higher permissiveness, as evidenced by increased E antigen expression and viral particle production, without compromising cell viability (>80%). Therefore, the K562 cell line was selected for subsequent experiments to determine whether its level of susceptibility and permissiveness is associated with deficiencies in the antiviral response.

### 2.2. DENV Progressively Induces the Expression of Genes in the IFN-I Pathway in Erythroid-Megakaryocytic Progenitor Cells

**Figure 2:**
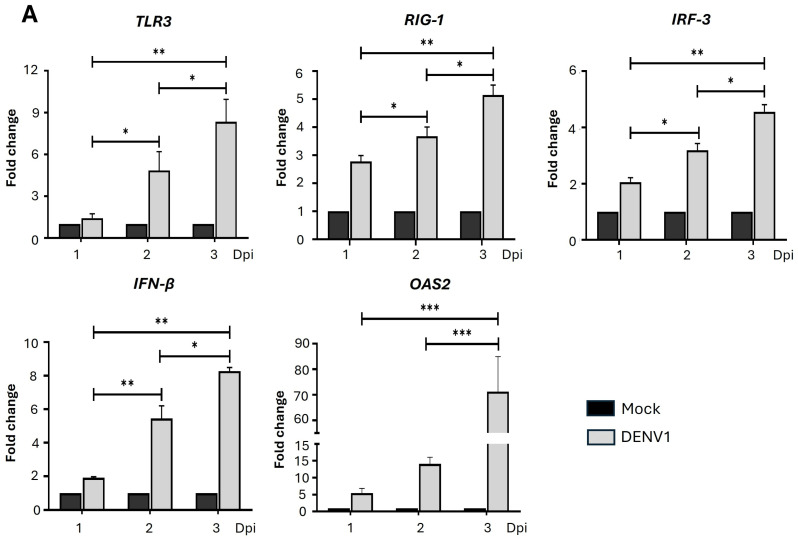

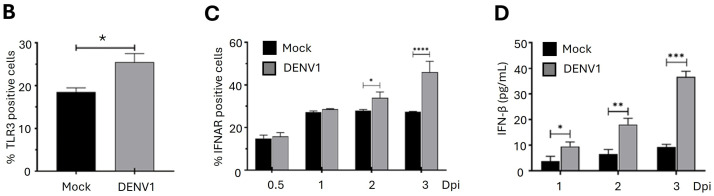
Expression of transcripts and proteins of the IFN-I pathway in K562 cells following infection with DENV1. (**A**) The relative expression of *TLR3*, *RIG-I*, *IRF3*, *IFN-β*, and *OAS2* transcripts was determined in K562 cells infected with DENV1 (MOI 0.5) at 1, 2, and 3 days post-infection (dpi). The analysis was performed using quantitative PCR (qPCR), with the ΔΔCt method and normalization to β-actin as the endogenous control gene. (**B**) Expression of TLR3 and (**C**) expression of IFNAR1 in K562 cells infected with DENV1 (MOI 0.5) at three dpi were assessed using monoclonal antibodies and flow cytometry. (**D**) IFN-β secretion expression in K562 cells infected with DENV1 (MOI 0.5) at 0.5, 1, 2, and 3 dpi by ELISA (enzyme-linked immunosorbent assay). Results are expressed as mean ± standard deviation from three independent experiments. Statistical differences were analyzed using two-way ANOVA followed by Tukey’s test (panels **A**,**C**,**D**), while a Student’s *t*-test was used to compare TLR3 expression (panel B). Statistical significance levels were represented as follows: *p* < 0.05 (*), *p* < 0.01 (**), *p* < 0.001 (***), and *p* < 0.0001 (****).

To characterize the intrinsic antiviral immune response of K562, the expression of key genes associated with DENV detection and activation of the type I interferon (IFN-I) pathway was evaluated. The relative expression of genes was evaluated in K562 cells infected with DENV1 up to three days post-infection (Figure 2A), using quantitative Polymerase Chain Reaction (qPCR). The results showed that all transcripts evaluated (*TLR3*, *RIG-1*, *IRF3*, *IFN-β*, and *OAS-2*) gradually increased their expression, reaching their maximum level on the third day. Among the transcripts evaluated, *OAS2* (2′-5′-oligoadenylate synthetase) stands out for achieving a 71-fold increase in expression compared to the non-infected condition (Figure 2A). In addition, the presence of the genes encoding the two subunits of the IFN-I receptor (*IFNAR1* and *IFNAR2*) was also evaluated, but only by endpoint PCR (Supplementary Figure S1). In this regard, we found an increase in the surface expression of TLR3 (Figure 2B) and IFNAR (Figure 2C) in K562 cells infected with DENV, as well as increased secretion of IFN-β (Figure 2D). Taken together, these findings indicate that cells of the erythroid-megakaryocytic lineage respond progressively to DENV by producing transcripts and proteins related to the antiviral response, particularly the type I interferon pathway.

### 2.3. DENV Promotes the Differentiation of Erythroid-Megakaryocytic Progenitors

**Figure 3:**
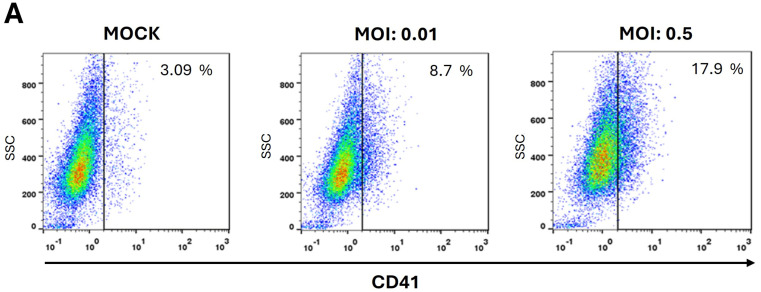

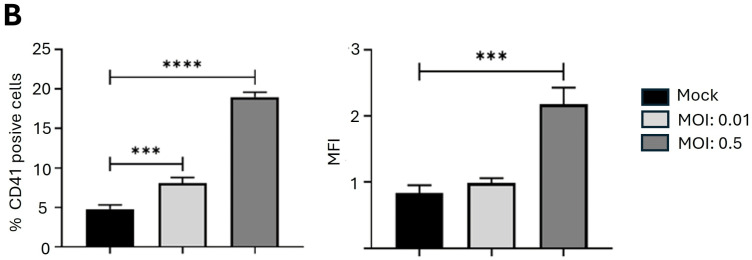
DENV1 induces CD41 expression in K562 cells. K562 cells were infected with DENV1 using an MOI of 0.01 or 0.5. Subsequently, three days post-infection (dpi), CD41 expression was evaluated using a monoclonal antibody and flow cytometry. (**A**) Representative dot plot image showing CD41 expression (*X*-axis) and Side Scatter (SSC, *Y*-axis). (**B**) Percentage of CD41-positive cells and mean fluorescence intensity (MFI) of the population. Bars represent the mean ± standard deviation of three independent experiments. Statistical analysis was performed using two-way ANOVA followed by Tukey’s test. Statistically significant differences are indicated as *p* < 0.001 (***), and *p* < 0.0001 (****).

Given that DENV1 infection of K562 cells induces a progressive expression of antiviral response components, and that certain viral infections can promote megakaryopoiesis by increasing the proportion of CD41^+^ cells—a characteristic marker of megakaryocyte maturation [26]—we evaluated whether DENV1 infection could induce the differentiation of K562 cells. The results indicate an increase in CD41 expression in DENV1-infected K562 cells at three days post-infection (dpi), an effect dependent on the multiplicity of infection (MOI) used (0.01 and 0.5) (Figure 3A,B). These findings suggest that DENV1 infection promotes erythroid–megakaryocytic precursor differentiation, potentially linked to the progressive activation of the antiviral response.

### 2.4. Differentiation of Erythroid-Megakaryocytic Progenitor Cells Regulates the Antiviral Response to DENV

**Figure 4:**
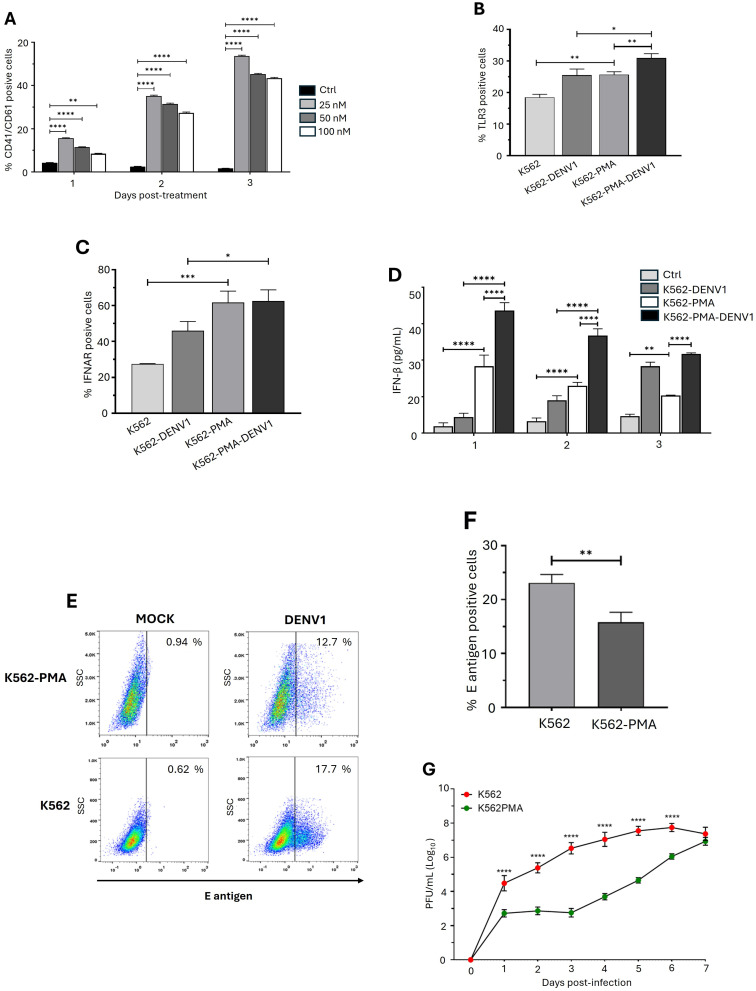
Effect of K562 cell differentiation on antiviral response during DENV1 infection. (**A**) Expression of CD41/CD61 in K562 cells following treatment with different concentrations of PMA (*Y*-axis) across multiple days. (**B**,**C**) Expression of TLR3 (**B**) and IFNAR1 (**C**) in K562 and K562-PMA cells infected with DENV1 at three days post-infection (dpi). (**D**) Kinetics of IFN-β secretion in K562 and K562-PMA cells, with and without DENV1 infection, quantified by ELISA. (**E**) Representative dot plots of K562 and K562-PMA cells infected with DENV1, showing E antigen detection by flow cytometry at three dpi. (**F**) Percentage of K562 and K562-PMA cells positive for DENV1 E antigen at three dpi. (**G**) DENV1 replication kinetics in supernatants from K562 and K562-PMA cells, determined by plaque formation assay (PFU/mL) over seven dpi. Panels (**A**–**C**,**E**,**F**) were analyzed by flow cytometry using monoclonal antibodies specific to the indicated antigens. In all infection experiments, a multiplicity of infection (MOI) of 0.5 was used. Statistical analysis was performed as follows: panel (**F**) was evaluated using Student’s *t*-test, while panels (**A**–**D**,**G**) were analyzed using two-way ANOVA followed by Tukey’s test. Results are presented as mean ± standard deviation from three independent experiments. The results are expressed as the mean ± standard deviation of three independent experiments. Statistical differences are indicated as *p* < 0.05 (*), *p* < 0.01 (**), *p* < 0.001 (***), *p* < 0.0001 (****).

To assess whether erythroid–megakaryocytic progenitor differentiation enhances the antiviral response, we employed a PMA stimulation model previously described by Kaur et al. [19]. Based on our viability assays (Supplementary Figure S2), the morphological changes observed (Supplementary Figure S3), and the increased expression of CD41 and CD61 (Figure 4A), we selected a concentration of 50 nM for 48 h to treat K562 cells (hereafter referred to as K562-PMA). Upon evaluating components of the type I interferon (IFN-I) pathway, we observed a higher percentage of TLR3-positive cells (Figure 4B) and IFNAR-positive cells (Figure 4C), along with increased IFN-β secretion (Figure 4D) in K562-PMA cells compared to unstimulated K562 cells. Notably, DENV-infected K562-PMA cells exhibited a higher percentage of TLR3^+^ cells and greater IFN-β secretion than uninfected K562-PMA cells (Figure 4B,D).

Once the K562-PMA differentiation model was established, we evaluated its impact on DENV infection. Susceptibility was first assessed by flow cytometry, quantifying the presence of the E antigen. A lower percentage of E antigen-positive cells was detected in K562-PMA cells compared to K562 cells (Figure 4E,F), although slightly higher than that observed in the MEG-01 progenitor (Supplementary Figure S4). Similarly, when analyzing cell permissiveness based on plaque-forming unit (PFU) production, we found that during the first four days post-infection (dpi), K562-PMA cells produced fewer PFUs than K562 cells (Figure 4G). However, viral particle counts reached comparable levels in the subsequent days. These findings underscore that the differentiation status of erythroid–megakaryocytic progenitors critically influences their antiviral capacity.

### 2.5. Differentiation of K562 Cells Increases the Expression of IRF3 and OAS2

**Figure 5:**
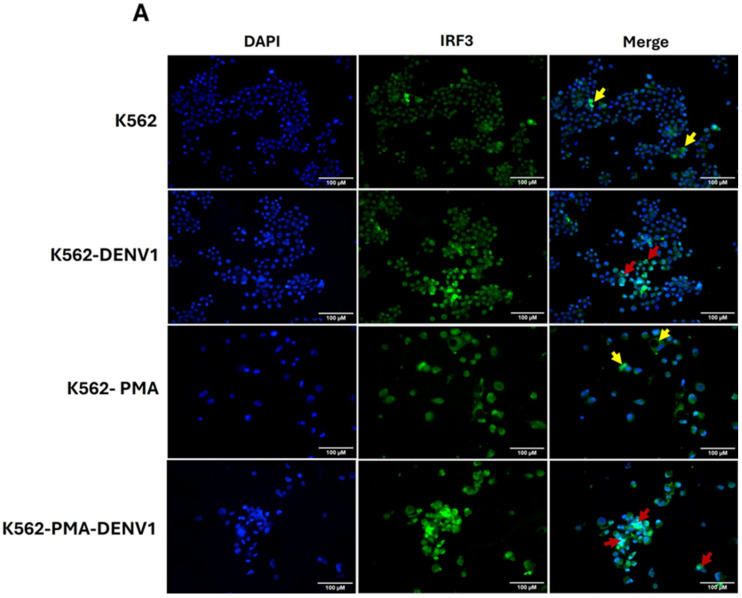

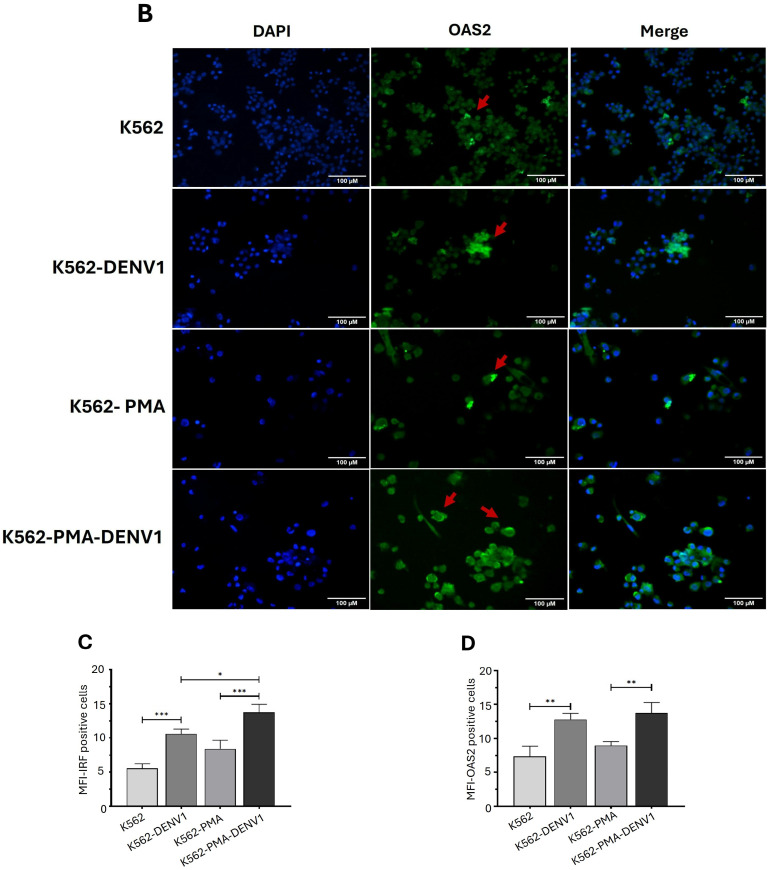
Expression of IRF3 and OAS2 in K562 and K562-PMA cells after infection with DENV1. (**A**,**B**) Cells were stained with monoclonal antibodies against IRF3 and OAS2 (green), and nuclei were counterstained with DAPI (blue). Samples were analyzed by fluorescence microscopy. In (**A**), yellow arrows indicate increased cytoplasmic expression of IRF3, while red arrows highlight enhanced nuclear localization of IRF3. In (**B**), red arrows indicate increased OAS2 expression. (**C**,**D**) Bars represent the mean ± standard deviation from three independent experiments showing the mean fluorescence intensity (MFI) of IRF3 (**C**) and OAS2 (**D**). Statistical analysis was performed using one-way ANOVA followed by Tukey’s test. Significant differences: *p* < 0.05 (*), *p* < 0.01 (**), *p* < 0.001 (***).

Considering the importance of the transcription factor IRF3 and the interferon-stimulated gene product OAS2 in the innate antiviral immune response [27,28], we evaluated the effect of K562 cell differentiation on the expression of these molecules using fluorescence microscopy. Our results clearly demonstrate that IRF3 and OAS2 expression is increased in K562-PMA cells compared to untreated K562 cells (Figure 5A–D). The most pronounced upregulation of IRF3 and OAS2 was observed in K562-PMA-DENV cells (Figure 5A–D). In Figure 5A, IRF3 shows enhanced nuclear localization, particularly in K562-DENV and K562-PMA-DENV cells. This evidence, together with the findings described in Section 2.4, supports the conclusion that erythroid–megakaryocytic progenitor differentiation is associated with a heightened antiviral response.

### 2.6. Exogenous IFN-β Enhances the Antiviral Response of Erythroid-Megakaryocytic Progenitors Against DENV in the Early Days of Infection

**Figure 6:**
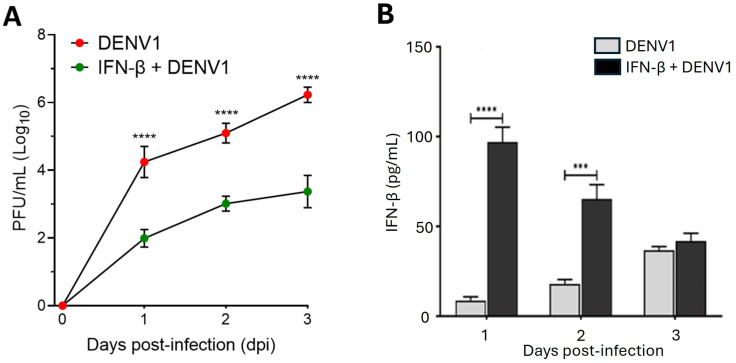
Pretreatment with IFN-β enhances the antiviral response in K562 cells. (**A**) Viral replication kinetics in supernatants from K562 cells pretreated with 100 U/mL recombinant IFN-β for 16 h and subsequently infected with DENV1 (MOI 0.5). Supernatants were collected every 24 h, and viral titers were quantified by plaque-forming unit (PFU) assay. (**B**) IFN-β secretion in K562 cells, either pretreated or not with exogenous IFN-β, was evaluated at 1, 2, and 3 days post-infection (dpi) using ELISA. Results are presented as mean ± standard deviation from three independent experiments**.** Statistical analysis was performed using two-way ANOVA with repeated measures, followed by Tukey’s test. Significant differences: *p* < 0.001 (***), *p* < 0.0001 (****).

Given the importance of IFN-β in limiting viral infections, we evaluated whether exogenous administration of this cytokine could enhance the antiviral response of K562 cells against DENV during the early stages of infection. To this end, cells were pretreated with 100 U/mL of recombinant IFN-β for 16 h prior to infection. Supernatants were collected every 24 h, and viral titers were quantified. The results revealed a significant reduction in viral replication from day one compared to untreated cells, and this reduction was consistently maintained over the subsequent two days (Figure 6A). Additionally, we observed that DENV1-infected K562 cells secreted higher levels of IFN-β when pretreated with the same cytokine (Figure 6B). These findings demonstrate that K562 cells respond to exogenous IFN-β by enhancing their antiviral response during the early phase of infection. The slow and limited endogenous secretion of this cytokine may be a key factor contributing to the increased permissiveness of erythroid–megakaryocytic progenitors to DENV infection.

## 3. Discussion

Our results confirm that the K562 and MEG-01 megakaryocytic progenitor cell lines are susceptible to DENV infection without compromising cell viability, as indicated by greater than 80% cell survival up to 7 days post-infection. However, K562 cells exhibited significantly higher levels of viral replication compared to MEG-01 cells. This finding aligns with the observations reported by Clark et al. (2016), who demonstrated that DENV-2 replicates efficiently in undifferentiated megakaryocytic progenitors (K562 and MEG-01), with viral loads comparable to those observed in Vero cells [18]. In contrast, our model revealed lower permissiveness to DENV1 in MEG-01 cells. This difference may be attributed to the viral serotype used, as both the serotype and genotype of DENV have been shown to modulate infection efficiency in the same cell type in vitro [17], or to a deficient antiviral response in the erythroid–megakaryocytic precursor, as demonstrated in this study. Regarding cell viability, our findings are consistent with studies in primary megakaryocytes, where DENV infection did not significantly reduce in vitro viability [11]. Taken together, these results support the notion that the erythroid–megakaryocytic progenitor (represented by the K562 cell line) is highly permissive to DENV and, according to current evidence, constitutes a principal target of DENV within the megakaryocytic lineage in the bone marrow [11,12,18,19]. Therefore, we consider it essential to characterize the mechanisms of the type I interferon (IFN-I) pathway in this cell lineage as part of the pathophysiological framework of dengue.

At the molecular level, the antiviral response in K562 cells was delayed. The induction of viral RNA sensors (*TLR3* and *RIG-I*), along with associated factors (*IRF3*, *IFN-β*, and *OAS2*), was low on the first day post-infection and reached peak expression by the third day. This delay in IFN-I pathway activation may explain the high permissiveness observed in this progenitor. Previous studies have shown that RIG-I, MDA5, and TLR3 act synergistically to restrict DENV infection and promote early IFN-β production [29,30]. In in vitro megakaryocytic models (MEG-01), DENV infection has been reported to induce elevated levels of RIG-I, MDA5, and TLR3 within 48–72 h, accompanied by increased IFN-β expression as early as the first day post-infection [21]—in contrast to the delayed response observed in K562 cells.

In DENV-infected K562 cells, delayed expression of TLR3 and IFNAR1 was observed, accompanied by a progressive increase in IFN-β secretion, which reached its peak concentration three days post-infection. These results indicate that, although K562 cells are capable of activating the type I interferon (IFN-I) pathway, they do so belatedly and less efficiently compared to myeloid lineages such as THP-1, which robustly secrete IFN-β from the first day post-infection (Supplementary Figure S5)—consistent with their monocyte phenotype, described as highly competent for IFN responses. In this context, it has been reported that K562 cells exhibit a deficiency in IFN-α production [31]. The above, combined with the low and delayed IFN-β secretion observed in our study, may contribute to the high viral permissiveness of this progenitor. Additionally, TLR3 has been described not only as an endosomal sensor of double-stranded RNA but also as an interferon-stimulated gene (ISG) [32], which aligns with the increased expression observed in our infected cells.

A notable finding of our study was the MOI-dependent increase in the expression of CD41 (integrin αIIb), a canonical marker of megakaryocytic differentiation. This observation supports the hypothesis that certain viruses can induce hematopoietic reprogramming toward specific lineages under stress conditions. For instance, in mice infected with the influenza A virus, emergency megakaryopoiesis has been reported, characterized by an increase in CD41^+^ cells committed to the megakaryocytic lineage [26]. In our model, the induction of CD41 expression by DENV in K562 cells suggests that the virus may promote differentiation of erythroid–megakaryocytic progenitors.

Megakaryocytic differentiation with PMA modulated the host–virus interaction in K562 cells (K562-PMA), as indicated by a reduction in initial susceptibility to DENV, a lower percentage of E antigen-positive cells, and viral replication during the first few days. These results are consistent with those reported by Banerjee et al. (2020), who found that PMA-differentiated MEG-01 cells were refractory to DENV infection [9]. However, viral titers in K562-PMA cells matched those in K562 cells in subsequent days, suggesting a transient restriction to infection and a loss of the antiviral state. This phenomenon may be attributed to viral antagonism mediated by specific nonstructural proteins such as NS2A and NS4B, which are known to block activation of the RIG-I/MAVS–TBK1/IRF3 signaling pathway, thereby inhibiting early IFN-β induction [33,34], or to IFN-I refractoriness mediated by USP18 [35]. Regarding TLR3 and IFNAR1, their basal levels were higher in K562-PMA cells, along with rapid and robust IFN-β secretion. In fact, IFN-β secretion by K562-PMA cells peaked on the first day post-infection and declined thereafter, following a pattern similar to that observed in THP-1 cells (Supplementary Figure S6). Altogether, these findings indicate that megakaryocytic maturation enhances antiviral responsiveness, akin to other cells of the innate immune system.

Finally, we found that pretreatment with recombinant IFN-β drastically reduced DENV replication from the first day and maintained an inhibitory effect throughout the course of the infection. These findings demonstrate that early activation of the type I interferon (IFN-I) pathway can counteract the high permissiveness observed in K562 cells [36]. Our results reinforce the notion that the low and delayed IFN-β response in erythroid-megakaryocytic progenitors is a key determinant of their susceptibility to DENV infection [22].

In conclusion, our data suggest that the maturation status of megakaryocytic progenitors critically regulates DENV infection. Undifferentiated K562 cells exhibit high susceptibility and an intrinsically delayed antiviral response, whereas differentiated cells (K562-PMA) show reduced initial infection and a more robust IFN-β response. This pattern supports the hypothesis that thrombocytopenia in dengue may result not only from peripheral platelet destruction but also from the infection of immature megakaryocytic precursors that lack an adequate antiviral defense.

## 4. Materials and Methods

### 4.1. Cultivation of K562 Cells and Differentiation with PMA

K562 cells (ATCC CCL-243, Manassas, VA, USA), of hematopoietic origin and with megakaryocytic potential, were cultured in RPMI 1640 medium (Roswell Park Memorial Institute; Biowest, Nuaillé, France) supplemented with antibiotic–antimycotic solution (Biowest, Nuaillé, France), L-glutamine (Biowest, Nuaillé, France), sodium bicarbonate (NaHCO_3_; Biowest, Nuaillé, France), and 5% fetal bovine serum (FBS; Biowest, Nuaillé, France). Cultures were maintained at 37 °C in a humidified atmosphere with 5% CO_2_. To induce differentiation into the megakaryocytic lineage, cells were treated with 50 nM phorbol 12-myristate 13-acetate (PMA; Sigma-Aldrich, St. Louis, MO, USA) for 48 h. Maturation was confirmed by increased expression of CD41 and CD61. Differentiated cells were designated as K562-PMA. MEG-01 (ATCC CRL-2021, Manassas, VA, USA) and THP-1 (ATCC TIB-202, Manassas, VA, USA) cells were maintained under the same culture conditions.

### 4.2. Virus

Dengue virus serotype 1 (DENV-1, American genotype; Western Pacific/74 strain, GenBank: AY145121.1) was used in this study. Viral stocks were propagated in Vero E6 cells (ATCC CCL-81, Manassas, VA, USA). Viral titers were quantified by plaque formation assay using BHK-21 (C-13) cells (ATCC CCL-10, Manassas, VA, USA), and results were expressed as plaque-forming units per milliliter (PFU/mL).

### 4.3. Viral Infection and Supernatant Collection

MEG-01, K562, K562-PMA, and THP-1 cell lines were infected with dengue virus at a multiplicity of infection (MOI) of 0.1 or 0.5, or as specified by the assay conditions. Viral adsorption was performed in 1 mL of serum-free RPMI 1640 medium (Roswell Park Memorial Institute; Biowest, Nuaillé, France) for 1 h at 37 °C, with gentle agitation every 15 min. Following infection, medium supplemented with fetal bovine serum (FBS; Biowest, Nuaillé, France) was added. Supernatants were collected on various days post-infection, clarified by centrifugation, and stored at −80 °C for subsequent viral titration. The volume extracted was replaced with fresh culture medium.

### 4.4. Flow Cytometry Analysis

K562 and K562-PMA cells were infected and incubated at various post-infection time points to evaluate the expression of viral envelope (E) protein and cellular receptors. Detection of the E protein was performed using the monoclonal antibody D1-4G2-4-15 (Novus Biologicals, Centennial, CO, USA), followed by Alexa Fluor^®^ 488-conjugated anti-mouse IgG secondary antibody (BioLegend, San Diego, CA, USA). For cellular markers, monoclonal antibodies conjugated against IFNAR-1 (APC; LSBio, Seattle, WA, USA), TLR3 (PE; BioLegend, San Diego, CA, USA), CD41 (FITC; BioLegend, San Diego, CA, USA), and CD61 (PE; BioLegend, San Diego, CA, USA) were used. Data acquisition was performed using a MACSQuant flow cytometer (Miltenyi Biotec, Bergisch Gladbach, Germany), and analysis was conducted with FlowJo software, version 10 (FlowJo, Ashland, OR, USA).

### 4.5. Detection of E Antigen, IRF3 and OAS2 by Immunofluorescence

MEG-01 and K562 cells were cultured on 12 mm glass coverslips pre-coated with 0.01% poly-L-lysine (Sigma-Aldrich, St. Louis, MO, USA) and infected with DENV1 at a multiplicity of infection (MOI) of 0.5 for 72 h. Cells were then fixed and permeabilized using Cytofix/Cytoperm™ solution (BD Biosciences, San Jose, CA, USA). Blocking was performed with Perm/Wash™ buffer (BD Biosciences, San Jose, CA, USA) supplemented with 1% bovine serum albumin (BSA; Biowest, Nuaillé, France). Detection of the viral envelope (E) antigen was carried out using the monoclonal antibody D1-4G2-4-15 (1:300; Novus Biologicals, Centennial, CO, USA), followed by Alexa Fluor^®^ 488-conjugated anti-mouse IgG secondary antibody (1:500; BioLegend, San Diego, CA, USA). Both antibodies were incubated for 1 h at room temperature, protected from light. For the detection of OAS2 and IRF3 proteins, primary monoclonal antibodies against OAS2 and IRF3 (both from Santa Cruz Biotechnology, Dallas, TX, USA) were used at a dilution of 1:200 under the same incubation conditions. Finally, coverslips were mounted with DAPI-containing mounting medium (NucBlue™, Invitrogen™, Carlsbad, CA, USA). Fluorescence images were acquired using a Leica DM2000 microscope (Leica Microsystems, Wetzlar, Germany) and analyzed with Leica Application Suite AF v3.1.0 and Fiji (ImageJ) v1.54p software.

### 4.6. Gene Expression Análisis

Total RNA was extracted from infected cells at different days post-infection and reverse transcribed to complementary DNA using Oligo(dT) primers and the RevertAid First Strand cDNA Synthesis kit (Thermo Scientific, Waltham, MA, USA). Gene expression was evaluated by endpoint PCR using GoTaq^®^ Hot Start Polymerase (Promega, Madison, WI, USA), and subsequently by real-time PCR (qPCR) using the Maxima SYBR Green/ROX qPCR Master Mix (2X) kit (Thermo Scientific, Waltham, MA, USA). The expression of the genes of interest was normalized with respect to the constitutive β-actin gene (Fw: GCGTTACACCCTTTCTTGAC, Rv: TTGTGAACTTTGGGG-GATGC). The TLR3 (Fw: CACCATTCCAGCCTCTTCGT, Rv: CA-GGGTTTGCGTGTTTCCAG), RIG-I (Fw: GTCCTCCTCTTTGCTGATCCC, Rv: ACCA-CACGTTGCTACACCAG), IRF3 (Fw: CGTGATGGTCAAGGTTGTGC, Rv: TGGGTGGCTGTTGGAAATGT), IFN-β (Fw: ACGCCGCATTGACCATCTAT, Rv: GTCT-CATTCCAGCCAGTGCT), OAS2 (Fw: TCTGCCTCCCATCCTACCAT, Rv: AACCAACC-CAGCTTCTGAGC), IFNAR1 (Fw: GTGATACACATCTCTCCTGG, Rv: GTATAATCC-CATTTAAGAACATAG) and IFNAR2 (Fw: GCTTTTGAGCCAGAATGCCT, Rv: CTCGTGTGTGCTTCTCCACT). The qPCR reactions were performed on a StepOne™ Real-Time PCR System (Applied Biosystems, Foster City, CA, USA) and analyzed using StepOne Software v2.3 (Applied Biosystems, Foster City, CA, USA).

### 4.7. Quantification of IFN-β

K562, K562-PMA, and THP-1 cells were seeded and infected with DENV as described. At defined time points post-infection, cell supernatants were collected, clarified by centrifugation, and stored at −80 °C until analysis. Detection and quantification of IFN-β were performed using a commercial Human IFN-beta DuoSet ELISA kit (R&D Systems, Minneapolis, MN, USA), following the manufacturer’s instructions. Absorbance readings were obtained at 450 nm with background correction at 570 nm using a Chromate™ microplate reader (Awareness Technology, Palm City, FL, USA). IFN-β concentrations were calculated from a standard curve generated using the kit’s reference controls.

### 4.8. Pretreatment with Recombinant IFN-β

K562 cells were pretreated with recombinant human IFN-β (Tonbo Biosciences, San Diego, CA, USA) at a concentration of 100 U/mL for 16 h prior to infection. Cells were subsequently infected with DENV at a multiplicity of infection (MOI) of 0.5 and incubated at 37 °C in a humidified atmosphere containing 5% CO_2_. Supernatants were collected at defined time points post-infection, clarified by centrifugation, and stored at −80 °C for viral titration. The extracted volume was replaced with fresh RPMI 1640 medium (Biowest, Nuaillé, France) supplemented with 5% fetal bovine serum (FBS; Biowest, Nuaillé, France) and antibiotic–antifungal solution (Biowest, Nuaillé, France).

## 5. Conclusions

Our results demonstrate, for the first time, that the erythroid–megakaryocytic precursor—represented by the K562 cell line—is highly permissive to DENV infection due to a delayed activation of the type I interferon (IFN-I) pathway, which is associated with its cellular maturation status. In this context, both the induced differentiation of K562 cells and their pretreatment with recombinant IFN-β enhanced the antiviral response. This in vitro model provides novel insights into dengue pathogenesis, particularly regarding its impact on megakaryocytic progenitors.

## Data Availability

The data presented in this study are available on request from the corresponding author.

## Supplementary Materials

The following supporting information can be downloaded at https://www.mdpi.com/article/10.3390/ijms262211081/s1.
