# Supplementary material for: Megakaryocytic Differentiation Regulates the Permissiveness and Antiviral Response of the Megakaryocytic Erythroid Progenitor to Dengue Virus
Source: Int J Mol Sci. 2025 Nov 16;26(22):11081. doi: 10.3390/ijms262211081 (PMC12652983; doi:10.3390/ijms262211081)
Supplement: Supplementary file 1 [file ijms-26-11081-s001.zip › ijms-3958628-supplementary.pdf]

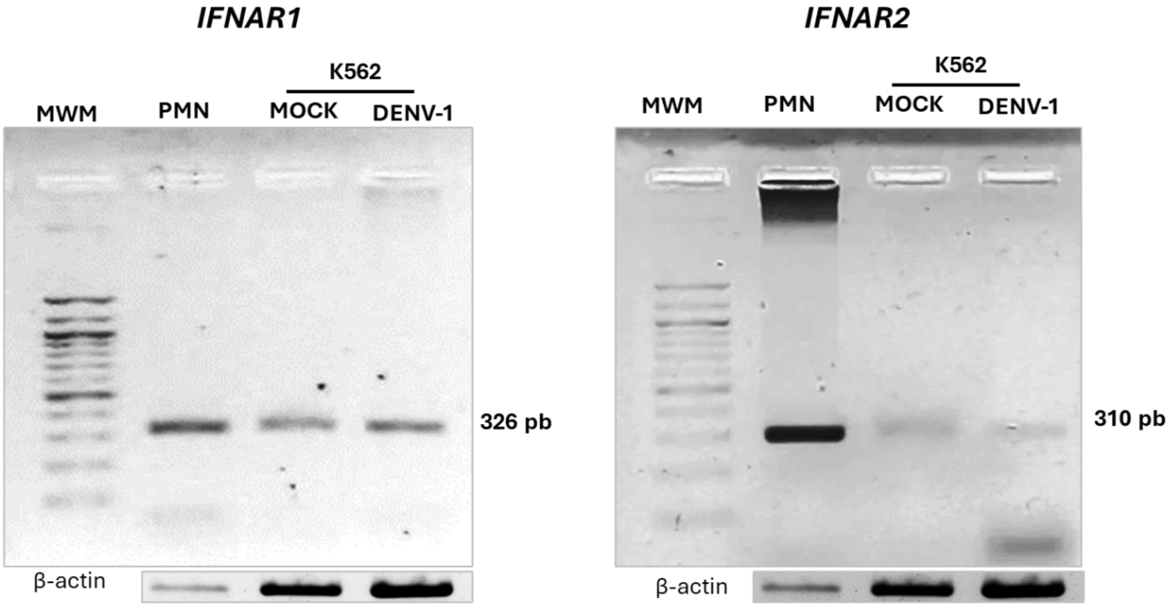

Figure S1. PCR amplification of type I interferon receptor subunits (IFNAR1 and IFNAR2) in K562 cells. The expression of type I interferon receptor subunits was evaluated by endpoint PCR using cDNA obtained from K562 cells. The amplified products corresponding to IFNAR1 (left) and IFNAR2 (right) were separated on 2% agarose gels stained with ethidium bromide (Sigma-Aldrich, St. Louis, MO, USA) and visualized under ultraviolet light using a Gel Doc™ XR+ photodocumenter (Bio-Rad, Hercules, CA, USA). A 100 bp DNA Ladder (Thermo Scientific, Waltham, MA, USA) molecular weight marker (MWM) was used to estimate the amplicon size. In both gels, lane 2 corresponds to polymorphonuclear cells (PMNs), included as a positive expression control. β-actin amplification is shown at the bottom of each gel and was used as an internal control to verify cDNA integrity and proper PCR performance. The β-actin images correspond to the uncropped original gels and represent the same experimental conditions used for IFNAR1 and IFNAR2. Together, the results confirm the presence of IFNAR1 (~326 bp) and IFNAR2 (~310 bp) transcripts in K562 cells.

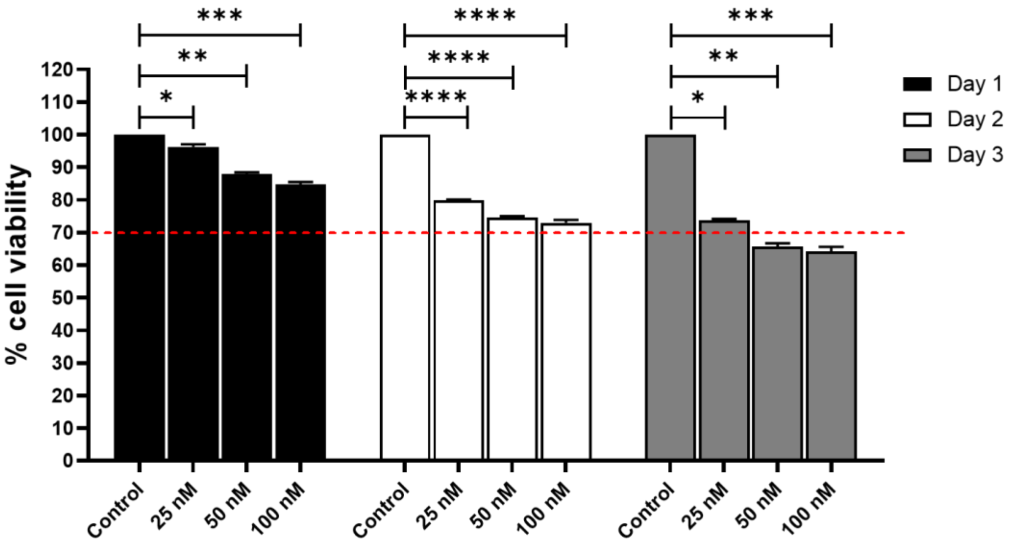

Figure S2. Evaluation of PMA cytotoxicity in K562 cells using the MTT assay. K562 cells were treated with increasing concentrations of phorbol 12-myristate 13-acetate (PMA; 25, 50, and 100 nM) and incubated for 24, 48, and 72 hours. Cell viability was determined using the MTT assay, and the results are expressed as the percentage of viable cells relative to the untreated control. The dashed red line indicates the 70% viability threshold, considered the lower limit of acceptable cytotoxicity. Exposure to PMA caused a time- and concentration-dependent decrease in cell viability, most evident at concentrations of 50 and 100 nM after 48 and 72 hours of treatment. The data represents the mean ± standard deviation of three independent experiments. Statistical analysis was performed using two-way ANOVA with Tukey's post hoc test. Significant differences: $p < 0.05$ (*), $p < 0.01$ (**), $p < 0.001$ (***), $p < 0.0001$ (****).

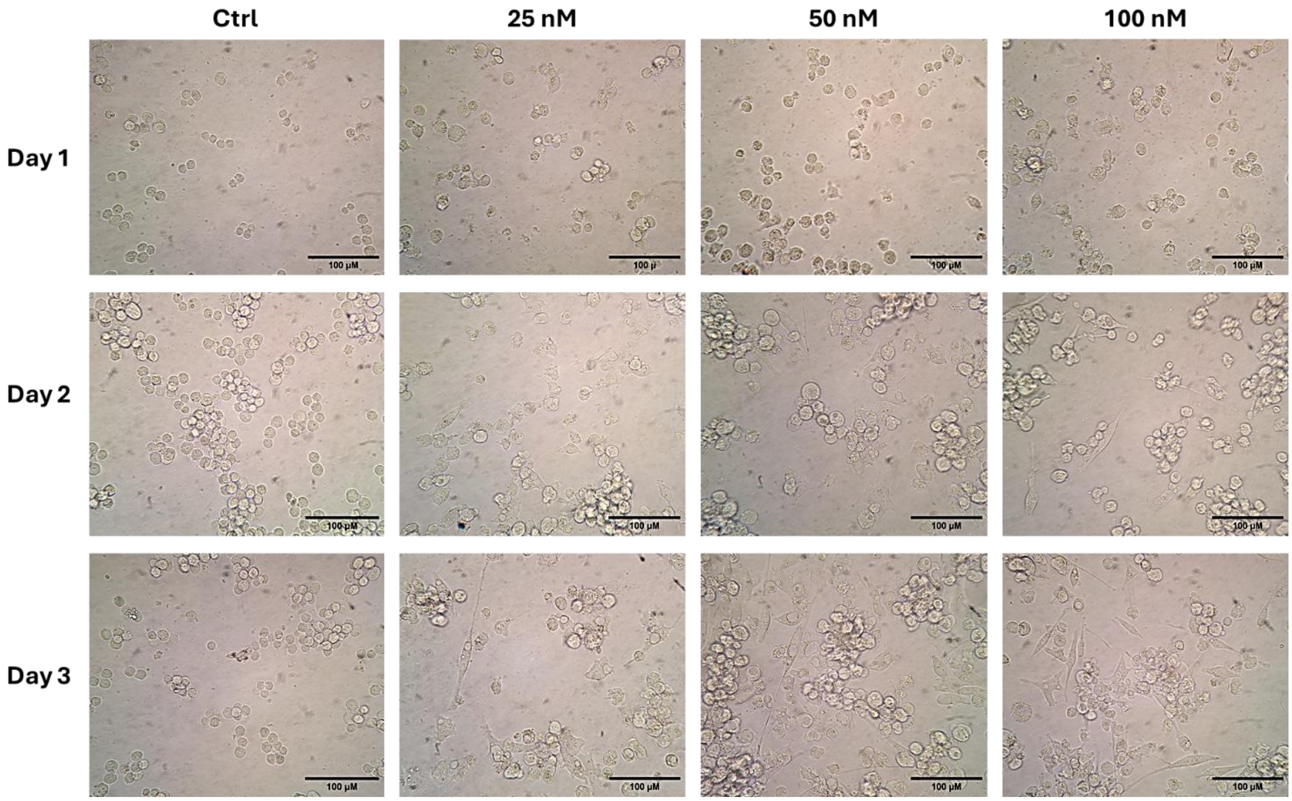

Figure S3. PMA-induced morphological changes in K562 cells. K562 cells were treated with increasing concentrations of phorbol 12-myristate 13-acetate (PMA; 25, 50, and 100 nM) and observed at 24, 48, and 72 hours under bright-field optical microscopy (10×). With increasing concentration and exposure time, the cells progressively transitioned from their rounded, non-adherent morphology to an elongated, spread-out shape. They tended to adhere to the substrate and appeared similar to fibroblast-type cells, characteristics compatible with a megakaryocytic maturation phenotype. The morphological change was most evident and sustained at 48 hours with 50 nM PMA and at 100 nM after 72 hours of treatment. Scale bar = 100 μm.

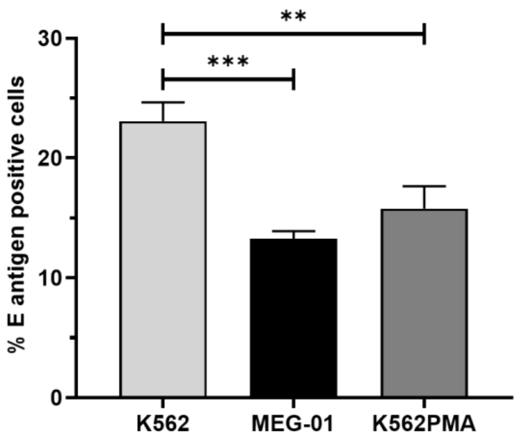

Figure S4. Comparison of dengue virus susceptibility among megakaryocytic lineage cells. K562, K562-PMA, and MEG-01 cells were infected with DENV1 (MOI 0.5) for 3 days and subsequently analyzed by flow cytometry to detect envelope (E) antigen expression. Results are expressed as the percentage of E antigen-positive cells relative to the total population. Data represents the mean ± standard deviation of three independent experiments performed in triplicate. Statistical analysis was performed using one-way ANOVA followed by Tukey's post hoc test. Significant differences: $p < 0.01$ (**), $p < 0.001$ (***).

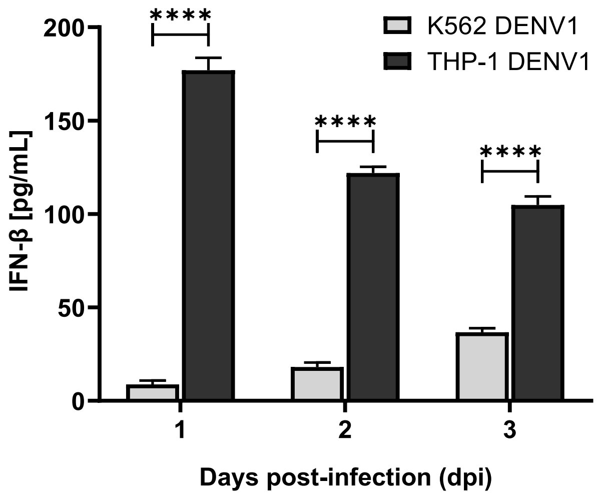

Figure S5. Comparison of DENV1-induced IFN-β secretion in K562 and THP-1 cells. K562 and THP-1 cells were infected with DENV1 (MOI 0.5), and supernatants were collected at 1, 2, and 3 days post-infection (dpi) to quantify IFN-β levels by ELISA. THP-1 cells showed significantly higher IFN-β secretion compared to K562 cells at all time points evaluated, reaching a maximum concentration at 24 h post-infection. Data represent the mean ± standard deviation of three independent experiments. Statistical analysis was performed using two-way ANOVA followed by Tukey's post hoc test. Significant differences: $p < 0.0001$ (****).

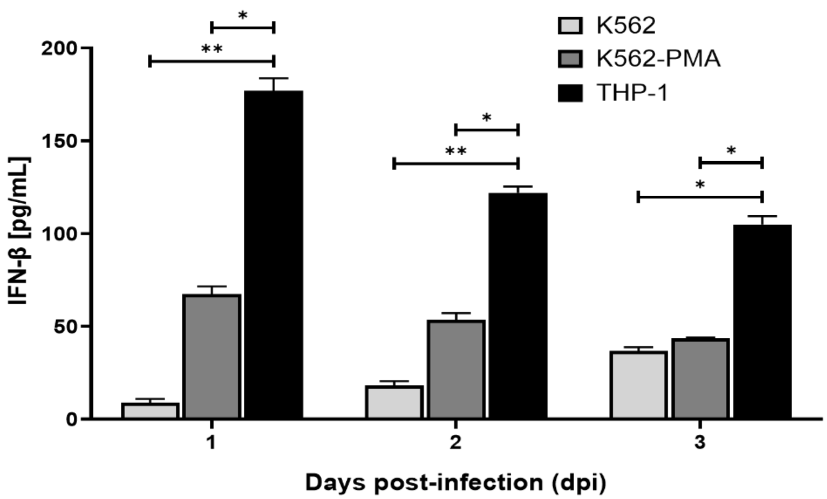

Figure S6. Comparison of DENV-1-induced IFN-β secretion in K562, K562-PMA, and THP-1 cells. K562, K562-PMA, and THP-1 cells were infected with DENV-1 (MOI 0.5), and supernatants were collected at 1, 2, and 3 days post-infection (dpi) to quantify IFN-β concentration by ELISA. Data are expressed as mean ± standard deviation from three independent experiments. Statistical analysis was performed using two-way ANOVA followed by Tukey's post hoc test. Significant differences: $p < 0.05$ (*), $p < 0.01$ (**).
